# An anatomical imposter in the left upper quadrant: Incidental beaver tail liver on chest computed tomography

**DOI:** 10.1016/j.radcr.2026.08.002

**Published:** 2026-08-28

**Authors:** Soheil Mirzaei, Zahra Motaghed, Reza Naseri, Pegah Hazrati, Farideh Shafeghat

**Affiliations:** a Department of Anatomy, Faculty of Medical Sciences, Tarbiat Modares University, Tehran, Iran; b Department of Radiology, Shahid Sattari Hospital, Shahid Beheshti University of Medical Sciences, Tehran, Iran; c Department of Radiology, Loghman Hakim Hospital, Shahid Beheshti University of Medical Sciences, Tehran, Iran; d Shahid Sattari Hospital Infectious Disease Specialist, Shahid Beheshti University of Medical Sciences, Tehran, Iran

**Keywords:** Beaver tail liver, Sliver of liver, Left hepatic lobe, Anatomic variant, Spleen, Computed tomography, Incidental finding

## Abstract

Beaver tail liver, also known as a sliver of liver, is a rare anatomic variant in which the left hepatic lobe extends laterally to the splenic region. Although usually asymptomatic, its imaging appearance may simulate perisplenic pathology and result in diagnostic confusion. A 55-year-old man presented to the infectious disease clinic with cough and dyspnea. Because of persistent respiratory symptoms and a history of tobacco use, non-contrast chest computed tomography was performed to evaluate possible pulmonary parenchymal abnormalities. On the lower sections of the scan, a structure with attenuation similar to that of the liver parenchyma was noted in the left upper abdominal quadrant adjacent to the spleen. Multiplanar reconstructed coronal and sagittal images demonstrated direct continuity of this structure with the left hepatic lobe, extending as a narrow tongue-like projection toward the spleen. These findings were consistent with beaver tail liver. No evidence of splenic injury, hematoma, perisplenic fluid collection, or focal mass was identified. The patient was managed conservatively for the respiratory symptoms, and no additional diagnostic or therapeutic intervention was required for this benign hepatic variant. Beaver tail liver is a rare but clinically important anatomic variant that may mimic perisplenic disease on imaging studies. Recognition of its continuity with the left hepatic lobe on multiplanar imaging and its similar attenuation to normal liver parenchyma is essential for accurate diagnosis and avoidance of unnecessary intervention.

## Introduction

Beaver tail liver, also known as sliver of liver, is a rare anatomical variant of the liver in which the left hepatic lobe extends unusually laterally, crosses the midline, and reaches the spleen; in some cases, it may come into contact with the spleen or partially or completely encircle it [[1], [2], [3], [4]] .The similar echogenicity of the liver and spleen on ultrasonography, as well as their similar attenuation on CT, may cause this variant to be mistaken for splenic subcapsular hematoma, perisplenic fluid collection, or splenic injury, particularly in the setting of abdominal trauma and FAST assessment [[1], [2], [3], [4], [5], [6]]. Therefore, awareness of this variant is of substantial importance for radiologists, emergency physicians, and surgeons in order to prevent misdiagnosis and unnecessary interventions [1,2,5].

## Case report

**Fig. 1:**
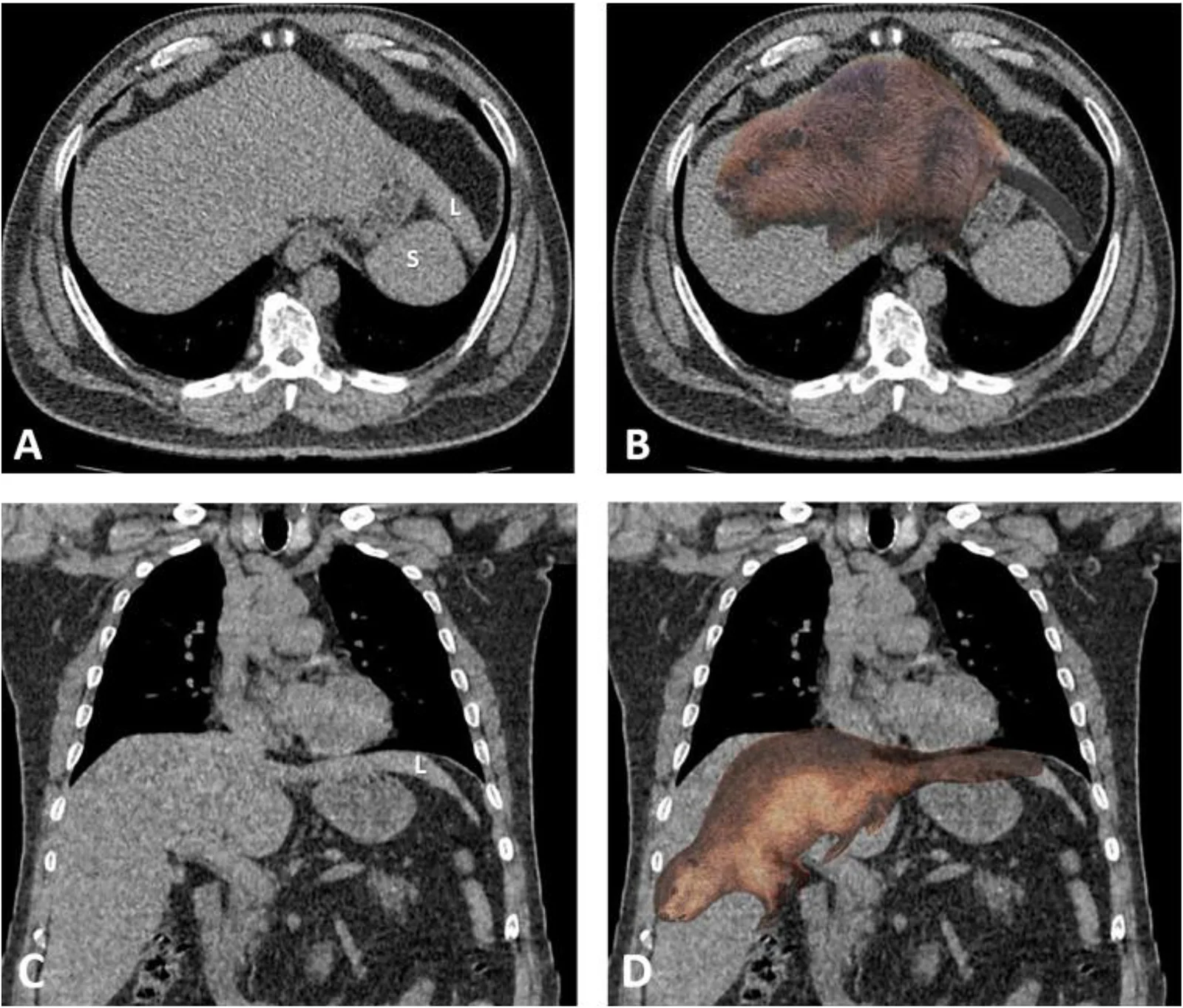
Incidental beaver tail liver on noncontrast chest CT. (A) Axial CT section shows the left hepatic lobe (L) extending laterally and posterior to the spleen (S), mimicking a perisplenic collection or subcapsular hematoma. Note that the attenuation of the extension is identical to the rest of the liver parenchyma. (B) An illustrative overlay on the axial image demonstrating the resemblance of the elongated left lobe to the tail of a beaver, providing the namesake for this anatomical variant. (C) Coronal reconstruction clearly depicts the direct continuity of the left hepatic lobe (L) as it tapers and wraps around the superior and lateral aspect of the spleen. (D) Coronal illustrative overlay highlighting the “beaver tail” morphology in the multiplanar view.

**Fig. 2:**
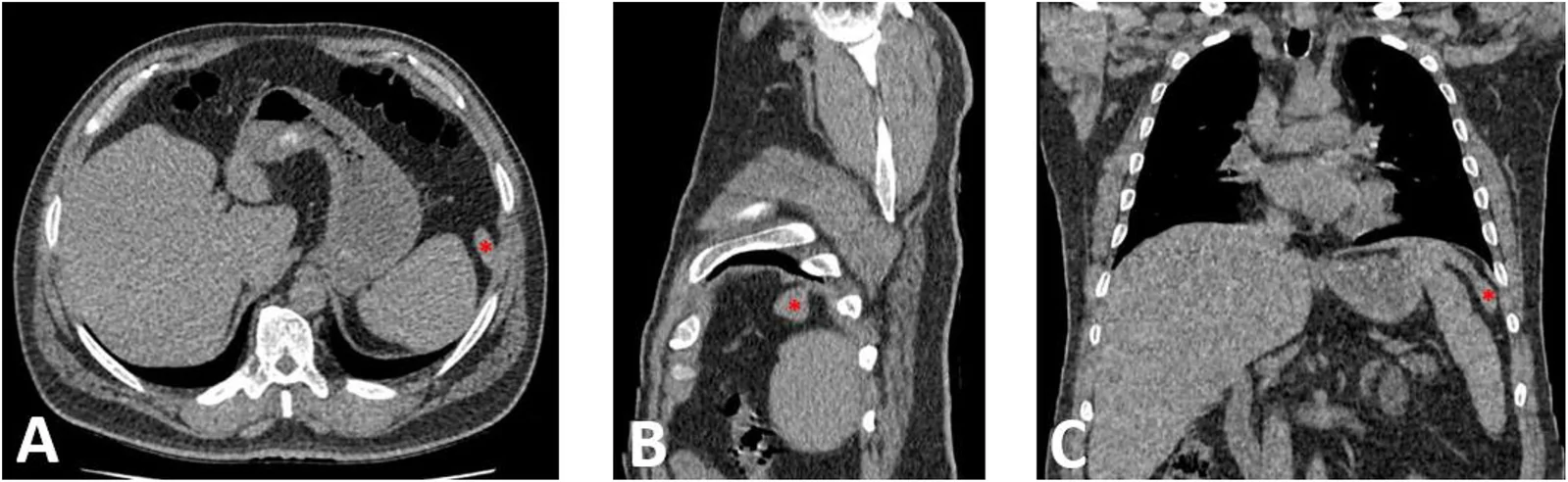
Noncontrast CT of the upper abdomen showing the elongated left hepatic lobe (beaver tail liver) mimicking a perisplenic mass. (A) Axial noncontrast CT image demonstrates the lateral tip of the elongated left hepatic lobe (red asterisk) wrapping around the spleen. The parenchymal attenuation of this extension is identical to the rest of the liver. (B) Sagittal and (C) coronal reformatted CT images clearly show the uninterrupted anatomical continuity of the tissue (red asterisks) with the left hepatic lobe, tracking superiorly and laterally along the splenic margin.

A 55-year-old man presented to the infectious disease clinic with complaints of cough and dyspnea. Given the persistence of respiratory symptoms and a history of tobacco use, a non-contrast chest computed tomography scan was requested to evaluate possible pulmonary parenchymal lesions. In the lower sections of the CT scan, a structure with attenuation similar to that of the hepatic parenchyma was incidentally observed in the left upper quadrant of the abdomen adjacent to the spleen. On axial images, this appearance could potentially be mistaken for perisplenic abnormalities, including subcapsular hematoma, perisplenic fluid collection, subphrenic abscess, or other masses in this region (Fig. 1, Fig. 2).

To determine the exact origin of this structure, the images were reconstructed and reviewed in the coronal and sagittal planes. These images demonstrated direct continuity of the aforementioned structure with the left hepatic lobe, extending as a narrow tongue-like projection toward the spleen. Accordingly, a diagnosis of beaver tail liver, or sliver of liver, was made as an anatomical variant of the left hepatic lobe. No evidence of splenic injury, hematoma, perisplenic fluid collection, or space-occupying lesion was identified in this region.

The patient received supportive management for his respiratory symptoms, including cough control, avoidance of triggering factors, and outpatient follow-up. Given the benign anatomical nature of the hepatic finding and the absence of any associated complication, no further diagnostic or therapeutic intervention for beaver tail liver was considered necessary.

## Discussion

### Definition and epidemiology

Beaver tail liver, or sliver of liver, is a rare anatomical variant of the liver in which the left hepatic lobe extends unusually laterally, crosses the midline, and reaches the vicinity of the spleen; it may come into contact with the spleen or partially encircle it [[1], [2], [3], [4]]. This variant consists of normal hepatic parenchyma and should be distinguished from an ectopic lobe or a pedunculated accessory lobe [1,2,7]. Epidemiological data regarding this condition are limited, and most available reports describe incidental findings identified during imaging examinations or anatomical studies [1,3,5,6].

### Imaging significance

The principal importance of this variant lies in its potential to cause diagnostic error on imaging, particularly in the evaluation of the perisplenic region [[1], [2], [3], [4],^7^]. Owing to the similar echogenicity of the liver and spleen on ultrasonography and their closely similar attenuation on CT, the elongated left hepatic lobe may be mistaken for splenic subcapsular hematoma, perisplenic fluid, subphrenic abscess, or splenic injury [1,2,5,7]. This issue becomes especially important in emergency settings, particularly in trauma patients, because it may lead to misinterpretation of FAST examinations and false-positive findings [1,5].

### Clinical presentation and literature review

Most cases of beaver tail liver are identified incidentally during the evaluation of unrelated conditions [[2], [3], [4]]. Reported cases have included adult patients presenting with abdominal pain, fever, hematuria, urinary tract infection, or trauma, as well as one rare case in a child with pneumonia [[1], [2], [3],^5^]. In most of these reports, the significance of the variant was not due to the production of direct symptoms, but rather to its potential for misinterpretation on imaging studies [2,3,5,7]. A related variant, referred to as hiding beaver tail liver, has also been described, in which the elongated portion of the left hepatic lobe is located on the medial side of the spleen and may make diagnosis more challenging [1,4].

### Differential diagnosis

The most important differential diagnoses include splenic subcapsular hematoma, perisplenic fluid collection, localized hemoperitoneum, subphrenic abscess, and splenic injury [1,2,4,5,7]. From an anatomical standpoint, this variant should be differentiated from Riedel’s lobe, accessory lobe, ectopic lobe, and the kissing sign [1,4,7,8]. Unlike the kissing sign, which usually occurs in the setting of hepatomegaly or splenomegaly, contact with the spleen in beaver tail liver results from focal extension of the left hepatic lobe [1,4].

Distinguishing a beaver tail liver from other abnormally positioned hepatic tissues is important to avoid misinterpretation on imaging. Accessory liver lobes may be pedunculated or sessile, remain continuous with the native liver, and may show a common capsule or characteristic biliary drainage, whereas ectopic liver tissue lies apart from the liver without parenchymal continuity [9,10]. Beaver tail liver, by contrast, represents an elongated left hepatic lobe extending toward or around the spleen and should not be confused with a separate accessory or ectopic hepatic mass [2,9]. In equivocal cases, multiplanar CT reconstructions and contrast-enhanced imaging can help confirm parenchymal continuity and vascular integration, which support the diagnosis and reduce the risk of labeling this normal variant as a perisplenic hematoma or splenic lesion [1,2].

### Key diagnostic points and clinical importance

The most important point in diagnosis is recognition of the continuity of the structure with the left hepatic lobe and identification of hepatic vessels or portal venous branches within it [1,2,5]. The use of Color Doppler and, in equivocal cases, CT can help differentiate this variant from hematoma or fluid collection [1,2,5,6]. Recognition of this variant is important not only for preventing imaging-related diagnostic errors, but also for avoiding inadvertent injury during surgical procedures and abdominal interventions, as this tissue may contain normal vessels and bile ducts [[2], [3], [4]].

## Conclusion

In summary, beaver tail liver is a rare but clinically important anatomical variant that should be considered, particularly during imaging evaluation of the perisplenic region and in trauma patients. Awareness of this condition, attention to its continuity with the liver, and the use of complementary modalities such as Color Doppler and CT can help prevent misdiagnosis, unnecessary procedures, and potentially hazardous interventions.

## Ethics statement

This case report was conducted in accordance with the ethical standards of the institutional and national research committee and with the 1964 Helsinki Declaration and its later amendments or comparable ethical standards.

## Patient consent

Written informed consent was obtained from the patient for publication of this case report and any accompanying images. A copy of the written consent is available for review by the Editor-in-Chief of this journal.
